# Highlight: Dosage-Sensitive Genes “Thread the Needle” of Selection

**DOI:** 10.1093/gbe/evaf144

**Published:** 2025-08-05

**Authors:** Pedro Andrade

Gene duplication, whether through whole-genome duplication or smaller-scale structural rearrangements, is a crucial mechanism that drives evolutionary novelty. The increase in the number of copies of a gene can result in relaxed selection on some of its copies, potentially enabling a change in function. However, some genes act in a dosage-sensitive way, which means that their phenotypic effects depend on how many copies exist in the genome; such dosage-dependent action constrains the evolution of copy number variation (Rice and McLysaght 2017). “Dosage-sensitive genes were previously shown to be resistant to gene duplications, except whole genome duplications, which automatically preserve ratios,” explains Aoife McLysaght, Professor at Trinity College Dublin in Ireland, who adds: “This constraint is especially true if the change affects a wide range of expressed tissues as it is more likely to upset an essential regulatory process,” reinforcing that many dosage-sensitive genes have been implicated in crucial developmental processes that, if disrupted, could result in disease or other disorders.

Does this mean that dosage-sensitive genes are excluded from evolutionary change of dosage or expression due to these constraints? Directly associating gene dosage and its regulatory effects at the genome scale has been challenging, but the recent development of large eQTL (expression quantitative trait loci) datasets allows researchers to investigate how specific loci influence the expression of one or more genes. This information is key to understanding how evolution of dosage-sensitive genes can bypass the deleterious effects of copy number variation, as McLysaght refers: “Tissue-specific regulatory variation offers the possibility of a more focused evolutionary change, as it could limit a change in expression regulation to one or a handful of tissues and crucially avoid interfering with critical regulation elsewhere.”

McLysaght and her team set out to investigate these questions on the evolvability of dosage-sensitive genes by analyzing human eQTL data gathered by the Genotype-Tissue Expression Consortium (GTEx Consortium 2017). They reported their findings in a new article published in *Genome Biology and Evolution* (Rice et al. 2025), with Alan M. Rice of the University College Dublin (at Trinity College Dublin and University of Bath, when the work was conducted) as first author. After identifying a set of candidate dosage-sensitive genes—duplicates resulting from whole-genome duplication, copy-number conserved genes, and haploinsufficient genes (those for which one copy is not enough to maintain function)—Rice et al. confirmed that this selection, in contrast to duplicates resulting from small-scale duplications, showed relatively higher between-tissue stability in expression and harbored a lower number of single-nucleotide variants overall. In other words, the authors found evidence of the dosage-sensitive genes having higher evolutionary conservation, both in their general activity and in their sequence itself.

While this might seem straightforward, a detailed inspection of the data revealed a curious pattern. Although dosage-sensitive genes were less influenced by eQTLs in each tissue, when the data were considered as a whole (i.e. pooling all tissues together), the dosage-sensitive genes showed a higher total number of regulatory effects than other genes. In other words, dosage-sensitive genes appear more likely to change function compared to other genes, an observation that is in contrast to our current understanding of the rules of molecular evolution. Furthermore, the eQTLs of the dosage-sensitive genes were significantly more distinct from each other across tissues than the eQTLs of other genes (which showed higher between-tissue similarity).

These observations suggested a specific hypothesis: dosage-sensitive genes evade selective constraints by tissue-specific fine-tuning of expression. This hypothesis is consistent with analyses that showed that dosage-sensitive genes had a lower proportion of tissues affected by each of their eQTLs, suggesting more tissue-restricted patterns of expression. Building on this, Rice et al. (2025) found that dosage-sensitive genes were less associated with eQTLs that acted broadly across tissues, and with those eQTLs associated with higher levels of expression. Thus, if a given genetic variant promotes the expression of a dosage-sensitive gene across many tissues, or has a high overall effect on its expression level, it is more likely to be purged by selection.

**Fig. 1.**
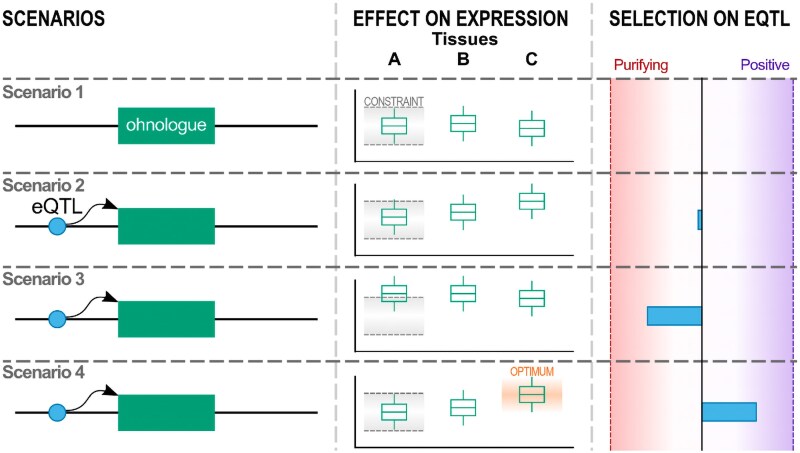
Conceptual model illustrating the effects of tissue-specific regulatory variants on the selective pressures and expression patterns of ohnologues, i.e. genes arising from whole-genome duplication and frequently subject to dosage constraints. When expression of an ohnologue is dosage-constrained in tissue A (and not necessarily in tissues B or C), maintaining expression levels across tissues resolves potential dosage-associated deleterious effects (scenario 1). If a mutation then creates an expression quantitative trait locus (eQTL) that affects the expression of the gene in tissue A, it should be subject to purifying selection, the strength of which depends on the magnitude of disruption to the gene's typical expression pattern (scenarios 2 and 3). In contrast, if the eQTL affects the expression of the ohnologue in a highly tissue-specific manner (scenario 4), and then the mutation can evade the effects of purifying selection if its expression in the other tissues is selectively neutral or beneficial. Figure provided by Alan M. Rice.

For McLysaght, the strong selective constraints on these genes force them onto narrow evolutionary paths. As she elegantly explains: “Imagine a dosage-sensitive gene that's expressed in three tissues, A, B, and C, and its expression in tissue A is highly constrained, with levels in tissues B and C being less critical. Evolutionary pathways that involve blunt changes in expression that affect all three tissues are ruled out as they are incompatible with the constraint on tissue A. Whole gene duplication is one of these “blunt” changes, because it should affect all tissues. However, pathways with changes in expression of just tissues B and/or C, but not A, can be explored.” In this way, dosage-sensitive genes are able to thread their way through a complex set of interactions as they evolve in response to selection in diverse tissues (Fig. 1).

The evolution of dosage-sensitive genes, while constrained, is a remarkably detailed example of how genomes leverage regulatory modularity to open up new evolutionary trajectories. These findings have implications that go beyond just understanding the effects of gene dosage itself, reinforcing our view that tissue-specific regulatory variation is a powerful instrument in the genome's toolkit for change. Given that many of the known dosage-sensitive genes in humans have been associated with developmental disorders, this study provides new perspectives on the mechanisms underlying disease. As McLysaght concludes: “If a given gene was known or presumed to be involved in disease in a dosage sensitive manner because of its association with pathogenic copy number variants, this study tells us that by drilling down into the details of eQTL and expression variation we may get an insight into the tissues where the disease phenotype arises.”

**Want to learn more?** Check out these other articles on gene dosage and expression recently published in *Genome Biology and Evolution*:

- Garber AI, Sano EB, Gallagher AL, Miller SR. Duplicate gene expression and possible mechanisms of paralog retention during bacterial genome expansion. Genome Biol Evol. 2024:16:evae089. https://doi.org/10.1093/gbe/evae089.
- Ostovar T, Landis JB, McCarthy EW, Sierro N, Litt A. Differential gene expression and unbalanced homeolog expression bias in 4 million-year-old allopolyploids of Nicotiana section Repandae. Genome Biol Evol. 2025:17:evaf029. https://doi.org/10.1093/gbe/evaf029.
- Taylor RS, Ruiz Daniels R, Macqueen DJ. Cell type resolved expression of duplicate genes retained from whole genome duplication in Atlantic salmon. Genome Biol Evol. 2025:17:evaf076. https://doi.org/10.1093/gbe/evaf076.
